# The Role of Gene Variants in the Iron Metabolism of Anemic Adolescent Girls

**DOI:** 10.7759/cureus.20128

**Published:** 2021-12-03

**Authors:** Sudarshan Reddy Varikuti, Devaraj J Parasannavar, Hemalatha Rajkumar, Tulja Bhukya, Uppala Satyanarayana, Manoj Kumar

**Affiliations:** 1 Clinical Epidemiology Division, Indian Council of Medical Research (ICMR) - National Institute of Nutrition, Hyderabad, IND; 2 Biochemistry Division, Dr. Pinnamaneni Siddhartha Institute of Medical Sciences, Vijayawada, IND; 3 Microbiology Division, Indian Council of Medical Research (ICMR) - National Institute for Research in Environmental Health, Bhopal, IND

**Keywords:** adolescent girls, snp, polymorphism, genetic variants, iron deficiency anemia

## Abstract

Background and objectives

Iron deficiency anemia (IDA) and the role of genetic variants in determining the iron status in adolescent girls are not yet well-understood. This study aims to investigate the association of the rs602662, rs1049296, rs1805051, rs855791, rs224589, and rs11568350 genes with IDA and iron bio-status parameters.

Methods

This study consisted of 132 patients (IDA group) and 110 healthy controls. The genotype was analyzed through polymerase chain reaction-restriction fragment length polymorphism.

Results

No differences were noted in the distribution of genotype and allele frequency single nucleotide polymorphism between the IDA and control group. In the IDA group, the GA carriers of rs602662 had a higher hemoglobin concentration (P=0.02) and packed cell volume (P=0.007), whereas transferrin saturation was increased in AA (P=0.02). The genetic variants rs1049296, rs1805051, rs224589, and rs855791 had a non-statistical significance on hematological parameters. Both the GT and TT carriers of the rs11568350 gene showed a low hemoglobin concentration (P=0.02 and <0.001) and mean corpuscular hemoglobin in GT carrier (P=0.01), whereas the TT risk of this gene showed a decreased packed cell volume (P=0.01). In the control patients, no association was observed with serum iron and hematological parameters.

Conclusion

Of these genetic variants, the GG and GA genotype frequency in rs602662 and the GG, GT, and TT in rs11568350 were associated with low iron status in anemic patients compared to the control patients.

## Introduction

Iron deficiency is one of the most important micronutrient deficiencies that affect people worldwide [1-2]. When an individual has low iron levels, it may lead to iron deficiency anemia (IDA). The prevalence of IDA among adolescent girls has been reported to be 56% [1], which may be due to inadequate dietary intake and genetic factors [3]. IDA affects nearly 58% of pregnant women and 50% of non-pregnant, non-lactating women [1]. Studies on the prevalence of anemia from different states of India reported the rates to be between 46%-98% [4-7].

Primary risk factors for IDA among adolescent girls include low dietary iron intake, defective absorption, poor bioavailability, worm infestation, and menstrual blood loss [8]. Genetic variants in iron metabolism have been shown to be associated with IDA [9-10]. However, the role of genetic factors in iron deficiency in adolescent girls in India is not known. It is known that adequate iron levels in the body are required for maintaining a normal healthy life and is necessary to tightly regulate these iron levels to avoid excess iron intake and iron toxicity [2].

A genome-wide association study has demonstrated the role of genetic components in determining IDA [11]. Gene polymorphisms encoding proteins have been reported to control several aspects of iron homeostasis, including transport (transferrin and lactoferrin), cellular uptake (transferrin receptor and human homeostatic iron regulator protein (HFE)), and TMPRSS6 (V736A) rs855791, which encodes the serine protease matriptase-6 and may regulate iron status in menstruating women [12]. A study from Spain observed that a large percentage of genetic variation of serum transferrin was explained by two single nucleotide polymorphisms (SNPs) located in the transferrin (TF) gene, which affects the iron transport and leads to iron deficiency anemia risk in menstruating women [12].

In another study, TMPRSS6 genetic variants were found to be strongly associated with serum iron, hemoglobin, mean corpuscular volume (MCV), and mean corpuscular hemoglobin (MCH) [13-14]. Subsequently, it was shown that serum iron was regulated by transferrin gene polymorphisms and red blood cell (RBC) count by transferrin receptor 2 variants [15].

A study completed by Lee et al. found that transferrin G277S mutation is a risk factor for IDA [16]. Another transferrin variant rs1049296, which is a missense variant occurs in the C-terminal lobe at position 570, with a proline replaced by a serine, affecting ferritin levels, serum iron, along with iron-binding capacity [17]. The rs1805051 gene polymorphism has been identified at nucleotide position 519 in exon 4, affecting transferrin binding [18]. The 1254T>C polymorphism in the exon region of the DMT1 rs224589 gene occurs within the coding region and may affect metal iron transport [19]. The ferroportin (Q248H) rs11568350 variant has the substitution of glutamine with histidine at position 248 (Q248H) in exon 6. In an African study, the ferroportin variant was seen as prudent protection against iron deficiency anemia [20]. The functional significance of the missense variant transferrin (G258S) (Gly) > (Ser) rs602662 polymorphism is still unclear [21]. The above studies found that these gene variants were associated with hematologic parameters, which may be relevant to IDA in India. Even though India continues to have the highest prevalence of IDA, there is limited information about the genetic aspects of IDA in this population.

This study aimed to investigate the association of six gene variants involved in iron metabolisms in an Indian adolescent girl population aged sixteen to nineteen years. There is a scarcity of data in this population to identify the role of iron polymorphisms on the hematologic characteristics between IDA and controls. The genetic variants considered for the study include rs602662, rs1049296, rs1805051, rs855791, rs224589, and rs11568350, and was conducted in a population living in the South Indian city of Hyderabad.

## Materials and methods

Participants and study setting

Our study presents a case-control design conducted in the urban areas of Hyderabad, India. The study was conducted by the Declaration of Helsinki and current Good Clinical Practice and was approved by the Institute Ethics Committee (ICMR-National Institute of Nutrition IEC number No: 08/II/2014). All participants were informed about the study details and written consent was given by each participant and their parents. A total of 242 female students aged 16 to 19 years were enrolled from two colleges. All selected volunteers were included in the study. However, the exclusion criteria were individuals with chronic diseases, those who were not menstruating regularly, those with other genetic abnormalities, such as hemoglobinopathy, individuals who donated blood, and mothers. Adolescent girls (n = 242) were recruited and their baseline iron status was evaluated. Based on the hematological parameters, participants were divided into the control (n = 110) and IDA (n = 132) groups. The participants with IDA were informed about their condition and subsequently treated with oral iron supplementation.

A questionnaire was used to collect socio-demographic information, menstrual history, family history of anemia, and clinical parameters. Anthropometric measurements were taken using standardized procedures. Bodyweight was measured using a calibrated Seca scale (to a precision of 100 g), and height was measured using a stadiometer incorporated into the scale (Seca, Germany). Body mass index (BMI) was calculated as weight/height squared (kg/m2). Blood samples of 5 mL each were collected by venipuncture and transferred to the laboratory via a cool box. Serum was separated by centrifugation at 1500× g for 15 min and stored at −80 °C for further analysis.

Hematologic profile

Parameters such as hemoglobin (g/dl), hematocrit (Hct, %), RBC, MCV (fL), MCH (pg), and mean corpuscular hemoglobin concentrations (MCHC, g/dl) of RBC were determined by ADVIA 120 automated hematology analyzer (Siemens Japan). Serum iron level was estimated using QuantiChrom™ iron assay kit (Bioassay Systems, Hayward, CA), total iron-binding capacity (TIBC, μg/dl) was measured by Biosystems (S.A Barcelona, Spain), and ferritin (ng/ml) was analyzed using EIA-1872 kit (DRG International Inc., Mountainside, NJ). Transferrin saturation (TS) was calculated by dividing the serum iron level by TIBC. IDA criterion was hemoglobin <12.0 g/dL, with serum ferritin <30 ng/mL and TS <15%.

Genetic polymorphism

DNA was extracted from whole blood using QIAamp DNA Blood Mini Kit (Qiagen, Hilden, Germany). Six genetic variants related to iron metabolism were identified from the literature, and genotyping was done via the polymerase chain reaction-restriction fragment length polymorphism. A summary of primers, polymerase chain reaction (PCR) conditions, endonucleases restriction, and sequencing conditions is provided in Appendix 1. Appendix 2 provides a summary of gene variants and the Human Genome Variation Society (HGVS) nomenclature and RefSeq ID annotations.

Statistical analysis

The chi-square (χ2) test p-value was used to verify whether genotype distributions were in the Hardy-Weinberg equilibrium. The results of genotype frequencies and allele frequencies were analyzed using multivariate logistic regression analysis. Statistical comparison of the demographic and hematologic characteristics between IDA and controls was performed using the student t-test. Association between the gene polymorphisms and hematological parameters was done using one-way analysis of variance, and Bonferroni post hoc analysis was used to compare the genotypes and hematologic characteristics. P<0.05 was considered statistically significant. Statistical analyses were done using IBM SPSS Statistics 22 (IBM Corp., Armonk, NY).

## Results

The mean (± standard deviation [SD]) age and BMI were comparable between both groups, and the average ages of the healthy and control groups were 17.6 ± 1.6 and 17.3 ± 1.4 years (P = 0.41), respectively, and their BMIs were 19.9 ± 4 and 19.2 ± 3.2 kg/m^2^ (P = 0.13), respectively. All hematological parameters were statistically significant between the IDA and healthy controls (P < 0.05) (Table 1).

**Table 1:** Age, BMI, and hematological parameters of the participants Data are mean ± standard deviation (SD), student’s t-test – comparisons between two groups (p < 0.05) is significant. Hb, hemoglobin; BMI, body mass index; PCV, packed cell volume; RBC, red blood cell; MCV, mean corpuscular volume; MCH, mean corpuscular hemoglobin; MCHC, mean corpuscular hemoglobin concentration; TS, transferrin saturation, TIBC, total iron-binding capacity

Parameters	IDA (N = 132)	Control (N = 110)	P value
Age in years	17.39 ± 1.42	17.67 ± 1.60	0.419
BMI in kg/m^2^	19.20 ± 3.21	19.90 ± 4.00	0.138
Hemoglobin (g/l)	102.7 ± 1.34	132.5 ± 0.76	<0.001*
PCV (%)	32.70 ± 3.11	39.25 ± 1.95	<0.001*
RBC (10^6^mm^3^)	4.25 ± 0.52	4.44 ± 0.41	0.002*
MCV (fl)	77.02 ± 10.33	85.86 ± 13.12	<0.001*
MCH (pg)	23.81 ± 3.65	33.96 ± 1.07	<0.001*
MCHC (g/dl)	28.34 ± 1.56	29.97 ± 2.77	<0.001*
Serum Iron (µg/dl)	29.99 ± 8.57	76.62 ± 26.99	<0.001*
Serum Ferritin (ng/mL)	14.35 ± 6.72	32.88 ± 14.13	<0.001*
Transferrin Saturation (TS %)	7.20 ± 3.88	17.95 ± 4.10	<0.001*
TIBC (µg/dl)	352.87 ± 45.83	289.20 ± 45.07	<0.001*

Table 2 shows the distribution of genotype frequency polymorphism of all the six genes; namely, rs602662, rs1049296, rs1805051, rs855791, rs224589, and rs11568350, which were studied between the IDA and control groups. The frequencies of none of these were significantly different between the IDA and control groups (P > 0.05). Further, we analyzed the association of genetic variants linked to iron metabolism with serum and hematologic parameters in both groups.

**Table 2:** Distribution of genotype frequency in rs602662, rs1049296, rs1805051, rs855791, rs224589, rs11568350 in IDA and controls OR, odds ratio; CI, confidence interval; IDA, iron deficiency anemia Logistic multiple regression test, chi-square (p < 0.05) significant

Gene variants	Genotype frequency	(N = 132)	(N = 110)	OR (95%CI)	P value
		IDA (N %)	Control (N %)		
rs602662	GG	81 (61%)	72 (65%)	1.05(0.74–1.48)	0.77
	GA	18 (14%)	11 (10%)		
	AA	33 (25%)	27 (25%)		
rs1049296	CC	94 (71%)	87 (79%)	1.60 (0.87–2.94)	0.12
	CT	34 (26%)	21 (19%)		
	TT	4 (3%)	2 (2%)		
rs1805051	AA	7 (5%)	12(11%)	1.54 (0.89–2.68)	0.12
	GA	101 (77%)	81 (74%)		
	GG	24 (18%)	17 (15%)		
rs855791	CC	30 (23%)	23 (21%)	0.80 (0.50–1.28)	0.35
	TC	83 (63%)	68 (62%)		
	TT	19 (14%)	19 (17%)		
rs224589	TT	81 (61%)	69 (63%)	1.06 (0.64–1.74)	0.81
	TC	35 (27%)	29 (26%)		
	CC	16 (12%)	11 (12%)		
rs11568350	GG	95 (72%)	81 (74%)	0.83 (0.51–1.37)	0.48
	GT	24 (18%)	17 (15%)		
	TT	13 (10%)	12 (11%)		

Table 3 shows the distribution of allele frequency, odds ratio, and confidence interval using multiple logistic regression analysis. In IDA participants, some of the studied polymorphisms, such as GA carrier for rs602662, showed a significant increase in hemoglobin (P = 0.02) and increased PCV (P = 0.007) compared with the GG carrier participants (Table 4).

**Table 3:** Distribution of allele frequency in rs602662, rs1049296, rs1805051, rs855791, rs224589, rs11568350 in IDA and controls OR, odds ratio; CI, confidence interval; IDA: iron deficiency anemia Logistic multiple regression test, chi-square (p < 0.05) significant

Gene variants	Allele frequency	(N = 132)	(N = 110)	OR (95%CI)	P value
		IDA (N %)	Control (N %)		
rs602662	G	180 (68%)	155 (70%)	0.76 (0.50–1.13)	0.32
	A	84 (32%)	65 (30%)		
rs1049296	C	222 (82%)	195 (89%)	1.06 (0.87–1.29)	0.53
	T	50 (18%)	25 (11%)		
rs1805051	A	115 (44%)	105 (48%)	0.92 (0.78–1.08)	0.31
	G	149 (56%)	115 (52%)		
rs855791	C	143 (54%)	114 (52%)	1.17 (0.98–1.39)	0.69
	T	121(46%)	106 (48%)		
rs224589	T	195 (74%)	167 (77%)	1.16 (0.85–1.59)	0.34
	C	67 (26%)	51 (23%)		
rs11568350	G	214 (81%)	179 (81%)	1.00 (0.89–1.13)	0.88
	T	50 (18%)	41 (19%)		

**Table 4:** Comparison of hematologic parameters of gene variants of rs602662 and rs1049296 One-way ANOVA model (post-hoc Bonferroni test) (p < 0.05) significant rs602662 (Pa = GG vs. GA) (Pb = GG vs. AA), rs1049296 (Pa = CC vs. CT) (Pb = CC vs. TT) ANOVA, analysis of variance

	IDA (N = 132)					Control (N = 110)				
rs602662	GG (n = 81)	GA (n = 18)	AA (n = 33)	P_a_	P_b_	GG (n = 72)	GA (n = 11)	AA (n = 27)	P_a_	P_b_
HB (g/L)	100 ± 15.3	109 ± 4.6	105 ± 9.6	0.02*	0.11	132 ± 7.7	131 ± 7.3	132 ± 7.9	1.00	1.00
PCV (%)	32.0 ± 3.5	34.50 ± 1.2	33.2 ± 2.2	0.007*	0.17	39.3 ± 1.92	38.4 ± 1.6	39.3 ± 2.1	0.51	1.00
RBC (10^6^ /µL)	4.2 ± 0.4	4.4 ± 0.3	4.1 ± 0.6	0.24	1.00	4.4 ± 0.42	4.2 ± 0.2	4.5 ± 0.4	0.65	0.91
MCV (fL)	75.9 ± 9.0	76.8 ± 7.4	79.7 ± 13.8	1.00	0.22	86.0 ± 14.43	90.0 ± 5.1	83.5 ± 11.4	1.00	1.00
MCH (pg)	23.5 ± 3.9	24.4 ± 2.8	24.2 ± 3.2	0.98	1.00	30.1 ± 2.73	30.7 ± 2.3	29.1 ± 2.9	1.00	0.28
MCHC (g/dl)	28.3 ± 1.5	28.1 ± 0.9	28.5 ± 1.7	1.00	1.00	33.9 ± 1.11	34.1 ± 0.8	33.9 ± 1.0	1.00	1.00
Ferritin (ng/ml)	14.5 ± 6.6	15.2 ± 7.0	13.4 ± 6.7	1.00	1.00	31.8 ± 13.75	37.8 ± 12.1	33.7 ± 15.8	0.56	1.00
Iron (μg/dl)	29 ± 9.2	30 ± 7.6	30 ± 7.3	1.00	1.00	77 ± 29.84	75 ± 17.3	73 ± 22.1	1.00	1.00
TIBC (µg/dl)	355.6 ± 47.5	348.6 ± 50.3	348.3 ± 39.3	1.00	1.00	290.7 ± 47.05	290.9 ± 32.1	284.4 ± 45.1	1.00	1.00
TS (%)	6.3 ± 3.5	8.4 ± 3.5	8.5 ± 4.3	0.11	0.02	18.2 ± 4.16	17.3 ± 4.7	17.4 ± 3.7	1.00	1.00
rs1049296	CC (n = 94)	CT (n = 34)	TT (n = 4)	P_a_	P_b_	CC (n = 87)	CT (n = 21)	TT (n = 2)	P_a_	P_b_
HB (g/L)	103 ± 13.2	102 ± 13.4	91 ± 19.2	1.00	0.27	132 ± 7.9	131 ± 6.9	135 ± 5.6	1.00	1.00
PCV (%)	32.8 ± 3.0	32.6 ± 3.0	30.5 ± 4.5	1.00	0.44	39.4 ± 2.0	38.5 ± 1.6	39.6 ± 1.1	0.26	1.00
RBC (10^6^ /µL)	4.2 ± 0.5	4.2 ± 0.6	3.8 ± 0.3	1.00	0.50	4.4 ± 0.4	4.2 ± 0.3	4.7 ± 0.4	0.23	1.00
MCV (fL)	77.0 ± 9.3	76.8 ± 12.9	78.4 ± 11.4	1.00	1.00	84.8 ± 14.2	90.0 ± 6.1	84.0 ± 9.9	0.32	1.00
MCH (pg)	24.0 ± 3.7	23.2 ± 3.2	23.5 ± 4.7	0.84	1.00	29.8 ± 2.7	30.7 ± 2.6	28.6 ± 3.7	0.47	1.00
MCHC (g/dl)	28.2 ± 1.5	28.6 ± 1.6	27.8 ± 0.6	0.52	1.00	33.9 ± 1.1	34.1 ± 0.8	34.0 ± 0.4	1.00	1.00
Ferritin (ng/ml)	14.6 ± 6.7	13.3 ± 6.6	15.5 ± 6.9	0.97	1.00	32.2 ± 14.5	35.3 ± 13.0	33.5 ± 0.5	1.00	1.00
Iron (μg/dl)	30 ± 8.5	29 ± 8.7	26 ± 8.2	1.00	1.00	76 ± 29.2	78 ± 17.2	76 ± 1.4	1.00	1.00
TIBC (µg/dl)	350.7 ± 48.0	353.0 ± 38.6	401.7 ± 19.3	1.00	0.08	287.4 ± 47.3	291.5 ± 31.1	341.5 ± 55.8	1.00	0.28
TS (%)	7.1 ± 3.8	7.8 ± 3.9	3.7 ± 0.9	0.92	0.27	17.9 ± 3.8	18.0 ± 4.9	18.2 ± 7.0	1.00	1.00

In IDA subjects, rs1805051 and rs855791 polymorphisms showed no statistically significant relation with hematologic parameters (Table 5).

**Table 5:** Comparison of hematologic parameters of gene variant rs1805051 and rs855791 One-way ANOVA model (post-hoc Bonferroni test) (p < 0.05) significant rs1805051 (Pa= AA vs. GA) (Pb = AA vs. GG); rs855791 (Pa= CC vs. TC) (Pb = CC vs. TT) ANOVA, analysis of variance

	IDA					Control				
rs1805051	AA (n = 7)	GA (n = 101)	GG (n = 24)	P_a_	P_b_	AA (n = 12)	GA (n = 81)	GG (n = 17)	P_a_	P_b_
HB(g/L)	91 ± 20.2	103 ± 13.2	101 ± 11.1	0.07	0.25	131 ± 8.2	132 ± 7.9	132 ± 5.7	1.00	1.00
PCV (%)	30.5 ± 4.3	32.8 ± 3.1	32.8 ± 2.5	0.17	0.23	39.2 ± 2.0	39.2 ± 2.0	39.1 ± 1.1	1.00	1.00
RBC (10^6^ /µL)	4.3 ± 0.2	4.2 ± 0.5	4.3 ± 0.4	1.00	1.00	4.4 ± 0.4	4.4 ± 0.4	4.4 ± 0.2	1.00	1.00
MCV (fL)	69.9 ± 7.3	77.6 ± 10.6	76.3 ± 8.9	0.16	0.43	83.4 ± 16.2	85.7 ± 13.8	88.0 ± 4.5	1.00	1.00
MCH (pg)	21.0 ± 3.9	24.0 ± 3.6	23.6 ± 3.5	0.10	0.26	29.8 ± 3.4	29.9 ± 2.8	30.0 ± 1.9	1.00	1.00
MCHC (g/dl)	28.5 ± 1.8	28.2 ± 1.4	28.5 ± 1.8	1.00	1.00	33.6 ± 1.3	34.0 ± 1.0	33.9 ± 0.9	0.83	1.00
Ferritin (ng/ml)	11.3 ± 6.4	15.0 ± 6.8	12.0 ± 5.6	0.44	1.00	31.2 ± 17.7	32.9 ± 13.8	33.8 ± 13.6	1.00	1.00
Iron (μg/dl)	26 ± 8.4	30 ± 8.5	29 ± 8.8	0.74	1.00	64 ± 25.5	77 ± 24.0	80 ± 38.6	0.36	0.36
TIBC (µg/dl)	365.7 ± 54.9	350.2 ± 44.6	360.0 ± 48.5	1.00	1.00	285.9 ± 59.9	286.5 ± 45.3	304.2 ± 28.6	1.00	0.84
TS (%)	4.5 ± 2.5	7.5 ± 3.9	6.5 ± 3.5	0.13	0.64	17.0 ± 4.0	18.0 ± 4.2	18.1 ± 3.6	1.00	1.00
rs855791	CC (n = 30)	TC (n = 83)	TT (n = 19)	P_a_	P_b_	CC (n = 23)	TC (n = 68)	TT (n = 19)	P_a_	P_b_
HB(g/L)	105 ± 10.5	103 ± 13.3	96 ± 16.9	1.00	0.10	133 ± 7.3	132 ± 8.1	131 ± 6.2	1.00	1.00
PCV (%)	33.3 ± 2.3	32.7 ± 3.1	31.4 ± 3.7	1.00	0.11	39.7 ± 2.1	39.0 ± 1.9	39.3 ± 1.8	0.34	1.00
RBC (10^6^ /µL)	4.3 ± 0.5	4.2 ± 0.5	4.0 ± 0.5	1.00	0.43	4.4 ± 0.4	4.4 ± 0.4	4.4 ± 0.3	1.00	1.00
MCV (fL)	76.9 ± 8.3	77.4 ± 10.5	75.3 ± 12.4	1.00	1.00	82.2 ± 16.3	86.4 ± 13.3	88.3 ± 5.8	0.56	0.39
MCH (pg)	24.1 ± 3.1	23.9 ± 3.7	22.5 ± 3.8	1.00	0.46	29.7 ± 2.7	30.1 ± 2.8	29.7 ± 2.6	1.00	1.00
MCHC (g/dl)	28.0 ± 1.4	28.4 ± 1.6	28.1 ± 1.4	0.73	1.00	33.9 ± 1.1	34.0 ± 1.0	33.6 ± 1.0	1.00	1.00
Ferritin (ng/ml)	14.5 ± 6.1	14.9 ± 7.1	11.4 ± 5.1	1.00	0.33	35.9 ± 15.4	32.8 ± 13.9	29.2 ± 13.0	1.00	0.39
Iron (μg/dl)	31 ± 7.7	29 ± 8.1	29 ± 11.5	1.00	1.00	76 ± 19.4	79 ± 30.2	68 ± 21.5	1.00	1.00
TIBC (µg/dl)	344.0 ± 53.6	353.0 ± 43.3	365.8 ± 42.3	1.00	0.32	286.4 ± 34.5	288.2 ± 49.1	295.8 ± 42.4	1.00	1.00
TS (%)	6.6 ± 3.7	7.3 ± 3.8	7.4 ± 4.5	1.00	1.00	17.3 ± 3.6	17.8 ± 4.4	18.9 ± 3.0	1.00	0.68

However, participants with GT genotypes had decreased hemoglobin and MCH in rs11568350 polymorphism (P = 0.02 and P = 0.01, respectively). The TT risk factor of this gene, however, showed decreased PCV (P = 0.005) and decreased hemoglobin (P ≤ 0.001) when compared to the GG genotype (Table 6).

**Table 6:** Comparison of hematologic parameters of gene variants rs224589 and rs11568350 One-way ANOVA model (post-hoc Bonferroni test) (p < 0.05) significant rs224589 (Pa = TT vs. TC) (Pb = TT vs. CC), rs11568350 (Pa = GG vs. GT) (Pb = GG vs. TT) ANOVA, analysis of variance

	IDA					Control				
rs224589	TT (n = 81)	TC (n = 35)	CC (n = 16)	P_a_	P_b_	TT (n = 69)	TC (n = 29)	CC (n = 11)	P_a_	P_b_
HB(g/L)	104 ± 13.4	100 ± 14.3	102 ± 11.5	0.44	1.00	132 ± 7.9	132 ± 7.9	133 ± 6.0	1.00	1.00
PCV (%)	33.0 ± 3.1	32.2 ± 3.3	32.1 ± 2.4	0.68	0.93	39.3 ± 1.9	38.9 ± 1.9	39.5 ± 2.0	1.00	1.00
RBC(10^6^/µL)	4.2 ± 0.4	4.2 ± 0.5	4.0 ± 0.6	1.00	0.19	4.4 ± 0.4	4.4 ± 0.4	4.3 ± 0.3	1.00	1.00
MCV (fL)	77.2 ± 10.6	75.2 ± 8.2	80.0 ± 12.3	1.00	0.95	86.1 ± 11.5	83.1 ± 17.9	90.5 ± 5.3	0.90	0.88
MCH (pg)	23.9 ± 3.7	23.3 ± 3.8	24.2 ± 2.9	1.00	1.00	29.8 ± 2.9	29.9 ± 2.2	30.9 ± 2.5	1.00	0.60
MCHC (g/dl)	28.3 ± 1.4	28.6 ± 1.8	27.5 ± 1.0	1.00	0.17	33.8 ± 1.0	34.1 ± 0.9	34.1 ± 1.2	0.56	0.99
Ferritin(ng/ml)	14.6 ± 7.0	14.7 ± 6.1	12.1 ± 6.0	1.00	0.56	33.1 ± 14.3	29.5 ± 10.5	39.1 ± 18.7	0.73	0.53
Iron (μg/dl)	32 ± 9.4	28 ± 8.0	29 ± 8.2	0.08	1.00	78 ± 30.3	75 ± 17.6	68 ± 25.1	1.00	0.77
TIBC (µg/dl)	352.8 ± 48.1	357.0 ± 41.6	343.8 ± 43.6	1.00	1.00	296.1 ± 44.6	278.2 ± 46.8	275.6 ± 37.7	0.21	0.43
TS (%)	7.6 ± 3.9	6.1 ± 3.4	7.3 ± 3.9	0.15	1.00	17.8 ± 4.0	17.6 ± 4.7	19.0 ± 2.5	1.00	1.00
rs11568350	GG (n = 95)	GT (n = 24)	TT (n = 13)	P_a_	P_b_	GG (n = 81)	GT (n = 17)	TT (n = 12)	P_a_	P_b_
HB(g/L)	105 ± 10.7	98 ± 15.9	90 ± 17.9	0.02*	<0.001*	133 ± 7.7	130 ± 6.6	129 ± 7.2	0.36	0.26
PCV (%)	33.2 ± 2.6	31.9 ± 3.5	30.3 ± 4.4	0.19	0.005*	39.4 ± 2.0	38.7 ± 1.5	38.7 ± 2.0	0.64	0.75
RBC (10^6^ /µL)	4.2 ± 0.5	4.2 ± 0.5	4.0 ± 0.5	1.00	0.45	4.4 ± 0.4	4.2 ± 0.4	4.4 ± 0.4	0.20	1.00
MCV (fL)	77.9 ± 9.6	74.4 ± 13.4	75 ± 8.3	0.43	1.00	85.8 ± 12.2	85.0 ± 19.5	87 ± 7.5	1.00	1.00
MCH (pg)	24.4 ± 3.4	22.1 ± 3.6	22.2 ± 3.7	0.01*	0.11	29.9 ± 2.7	30.3 ± 2.4	29.4 ± 3.2	1.00	1.00
MCHC (g/dl)	28.3 ± 1.5	28.5 ± 1.4	27.9 ± 1.5	1.00	1.00	34.0 ± 1.0	33.7 ± 1.1	33.7 ± 1.0	1.00	1.00
Ferritin (ng/ml)	14.8 ± 6.6	13.6 ± 6.9	12.2 ± 6.8	1.00	0.60	33.2 ± 14.5	29.0 ± 12.9	35.9 ± 13.2	0.78	1.00
Iron (μg/dl)	30 ± 7.8	27 ± 9.6	30 ± 11.6	0.57	1.00	78 ± 29.4	70 ± 12.7	71 ± 23.8	0.71	1.00
TIBC (µg/dl)	348.5 ± 47.2	362.5 ± 40.6	366.2 ± 42.2	0.54	0.58	288.7 ± 48.6	300.5 ± 34.2	276.1 ± 29.2	0.98	1.00
TS (%)	7.5 ± 3.8	6.9 ± 3.7	5.4 ± 4.06	1.00	0.21	17.8 ± 4.1	17.3 ± 3.9	19.6 ± 4.2	1.00	0.44

Furthermore, the control participants did not show any association between gene variants and hematologic parameters.

## Discussion

In our study, 55% of participants were iron deficient, which is almost similar to previously reported values 56% [1]. The mean hematological parameters, including PCV, RBC, MCV, MCH, and MCHC, were significantly lower in participants with IDA than in controls. Serum iron biomarkers were also significantly lower in all participants with IDA than in controls. The prevalence of IDA in the population of women in India is 53% from 15 to 49 years [22]. Our study observed a similar result for the analyzed age group, 16 to 19-year-olds). Furthermore, serum ferritin was lower in IDA individuals compared to the control, which is corroborated by a previous study where they confirmed serum ferritin to be one of the most specific markers for the assessment of iron levels and the detection of mild iron depletion [23].

The main findings of this study showed that the genetic variants rs602662 and rs11568350 affected some hematologic parameters. Specifically, the genotype frequency GG and GA carriers in rs602662 resulted in a reduction in HB and PCV levels, both of which were statistically significant. This is unsurprising since rs602662 is the main polymorphism in iron metabolism. For the same gene variant, a homozygous comparison between GG and AA carriers led to a significant reduction in TS levels. As a result, this gene variant may have a role in IDA development. Moreover, for the genetic variant rs11568350, both homozygous and heterozygous carriers experienced a reduction in HB levels. Only the carriers of TT had a reduction in PCV levels. All other hematologic parameters were not significant for the rs11568350 genetic variant. Genotypic and allele frequencies for the rs602662 and rs11568350 variants showed variations among IDA groups. Therefore, these polymorphisms could impact the prevalence of IDA.

The rs1049296 variant is a missense variant of the transferrin gene [16] that does not affect the parameters in iron metabolism as our results were not statistically significant, which is supported by a previous study [24]. Although the rs1805051 gene polymorphism has been reported to affect transferrin receptor function [25], in our study, none of the hematological parameters were significantly affected in the heterozygous and homozygous participants with IDA compared with the control, suggesting that this polymorphism may have little to no role in the development of IDA. In contrast to previous studies where the genetic variant rs855791 was significantly associated with ferritin levels and anemia in adolescent girls [26] and reduced serum iron levels [13-14], our study did not validate these findings, as the allele frequencies did not statistically affect any hematological parameters. Additionally, despite other studies showing the role of rs11568350 polymorphism in ferroportin (FPN1) and DMT1 rs224589 [27-28] in iron status, neither of these gene variants had a significant effect on iron metabolism in IDA participants compared with the control.

## Conclusions

In conclusion, there are significant gaps in the frequencies of genetic markers of the various populations; various gap-filling tests on the chosen population would make it conceivable to utilize the current perceptions more effectively in developing prophylaxis for a population with high frequencies of IDA, with FPN1 rs11568350 and rs602662 being associated with low iron status in anemic subjects and the predisposition to IDA in adolescent girls. There is yet a requirement for the assimilation of the new outcomes and additional investigations. The study has the limitation of being conducted in a single center and limited sample size. The sample for the control group should be expanded in the next analysis, along with the expansion of the age group to incorporate a better sample size that reflects the general population. Although a further study in a larger population is required to determine reliability, this study also has public health suggestions, as it demonstrates the genetic factors involved in the etiology of iron deficiency anemia in the adolescent girl population. As different examiners had genomic variety among the various populations around the world, some of the associations between polymorphisms and iron status previously reported for other populations were not reproduced in our study. This variation may be due to heterogeneity in genetic makeup between populations; population-specific genome studies should be conducted rather than inferring genetic data across populations. Taken together, our study highlights a link between IDA and genetic polymorphisms in a specific adolescent girl population.

## Appendices

Appendix 1

**Table 7:** Primer sequences and PCR conditions PCR, polymerase chain reaction

Generic Name	RefSeq ID		Fragment size	Enzyme
TF (G258S)	rs602662	CCCAGAGAACTTGGCAAACA	133	Alu1 (112+21)
		TCCCAGATCAAGTCCTCCTAGC		
TF (P570S)	rs1049296	GCCTTGACGGTACCAGGTAA	180	BstE2 101+46+36)
		CATACCTGCTGTTGACGTAATATCTT		
TR (S82G)	rs1805051	GGGATGACCTGAAGAGAAAGTTGTC	254	Ban1 (200+54)
		GTTCTACCTTTTCCCCTACCAGTATAGTTT		
TMPRSS6 (V736A)	rs855791	TAGAGAACAGGGGCTCCAGG	249	Stu I (125)
		ATGTGGGCAGCATCCTTTC		
DMT1 (1254T/C)	rs224589	CTTTGCCCGAGTGGTTCTGACTCGCTCGAT	229	MboI (198 + 29)
		TTCCTCTCAATATCCCCC		
FPN1 (Q248H)	rs11568350	CGTTCTGCTCTGGAAGGTTT	147	Pvu II
		GGTCTGAACATGAGAACAAAAGG		

Appendix 2

**Table 8:** Summary of gene variants and Human Genome Variation Society (HGVS) nomenclature and RefSeq ID annotations

HGVS Nomenclature		Generic Name	RefSeq ID
NC_000019.10: g.48703728G>A		TF (G258S),	rs602662
NC_000003.12: g.133775510C>G		TF (P570S),	rs1049296
NC_000003.12: g.196052101C>T,		TR (S82G),	rs1805051
NC_000022.10: g.37462936A>G		TMPRSS6 (V736A)	rs855791
NC_000012.12: g.51005267T>A		DMT1 (1254T/C)	rs224589
NC_000002.11: g.190430096C>A		FPN1 (Q248H)	rs11568350
